# What Is Secondary about Secondary Tropical Forest? Rethinking Forest Landscapes

**DOI:** 10.1007/s10745-020-00203-y

**Published:** 2020-12-14

**Authors:** Adam Pain, Kristina Marquardt, Arvid Lindh, Niles J. Hasselquist

**Affiliations:** 1 Department of Urban and Rural Development, Swedish University of Agricultural Science, 750 07 Uppsala, Sweden; 2 Department of Forest Ecology and Management, Swedish University of Agricultural Science, 90183 Umeå, Sweden

**Keywords:** Secondary forest, Forest landscapes, Disturbance, Regeneration, Public authority, Subsistence livelihoods

## Abstract

Forests have long been locations of contestation between people and state bureaucracies, and among the knowledge frameworks of local users, foresters, ecologists, and conservationists. An essential framing of the debate has been between the categories of primary and secondary forest. In this introduction to a collection of papers that address the questions of what basis, in what sense, and for whom primary forest is ‘primary’ and secondary forest is ‘secondary,’ and whether these are useful distinctions, we outline this debate and propose a new conceptual model that departs from the simple binary of primary and secondary forests. Rather, we propose that attention should be given to the nature of the disturbance that may alter forest ecology, the forms of regeneration that follow, and the governance context within which this takes place.

## Introduction

There have been two long running but somewhat separate debates concerned with the ‘rural’ and its transitions that need to be better connected. The older of the two has been concerned with the role of small-scale family farms and their eventual fate. While models of agrarian transition have aspired to and predicted the demise of a peasant economy that has persisted into the twentieth-first century, such that most farms in the global south are small family-operated smallholdings (Wiggins 2006). The majority of these smallholders are poor (Boltvinik and Mann 2016) and are likely to remain so given the evidence of blocked agrarian transitions (Bernstein and Byres 2002). There has also been resistance to the inevitability of incorporation into the legal global commodity markets reflected both in the rise of peasant movements (Borras *et al.* 2008) and in the cultivation of narcotic crops (Pain and Hansen 2019:257). But there is a growing ‘surplus’ in the landless rural population in some countries with limited futures in either the agrarian or urban economy (Li 2014). While mainstream rural development narratives remain focused around opportunity, competition, entrepreneurship, and value chains (see IFAD 2016), much less visible are rural policies addressing the environment, climate change, and alternative approaches to secure subsistence economies and perhaps something more enduring for rural small-scale farmers.

The second debate has been centred on forests and their management. Historically forest policy has focused on production, conservation, catchment integrity, and revenue (a ‘forestry without people’ perspective). Over time forest policy has shifted and has given some recognition to rural users and allowed forms of co-management and benefit sharing (e.g., community forestry). Nevertheless, a deep hostility to what are seen to be agricultural land management practice with trees (e.g., swidden agriculture) in tropical forests has persisted. Consequently, the rise of the global environmental agenda has repositioned forestry in a conservation agenda and the need to preserve primary tropical forests for the global good. This in turn has brought notions of forest transition (Mather and Needle 1998) to the fore in policy thinking to justify and legitimate forest management practices including the re-emergence of fortress forestry, albeit married to the commodification of forests through programmes such as REDD+ (Leach and Scoones 2013).

Forestry as a knowledge system and practice in the global south has long been contested. It has its origins in the ways in which European colonisation created a regime of categories of land use that delineated agriculture from forests and defined what constituted proper management of each. These categories fitted with the colonial imperatives of how things should be ordered, as Bhattacharya (2018) has shown in the case of the conquest of the Punjab in India. Here a priori concepts framed as specific land use types were mapped to a highly filtered vision of what could be seen on the ground even if these categories did not fit with what was actually there. It was only through the coercive power of the colonial state that a new rural landscape could be created. This colonial shaping of the rural world has left deep institutional legacies in terms of organisation and the knowledge frameworks that have structured the agricultural and forestry sectors.

These knowledge frameworks are increasingly challenged, both in agriculture (Sumberg and Thompson 2012) and in forestry. An early marker of the forest debates can be found in questions surrounding who decides what a forest is (Thompson and Warburton 1986). There is also a long history of scholarship questioning the belief in pristine forest landscapes, the notion of foresters as experts, and the limits and deficiencies of forest management practices (Sunseri 2009; Mathews 2011; Hansen and Lund 2017). Although forest policy can be seen as high-modernist and authoritarian (Scott 1998), there are strong grounds to challenge and rethink the ability of normative forestry models (Lund 2018) to address the complexity of the ecological and social life of forests (Hecht *et al.* 2014).

Underlying the long running contention between forestry practices and the populations that live in or near them and make use of their resources (Thompson 1975) are questions of land and the law and the nature of forests as territory and property. As Lund (2016:1199) has argued, the political authority of the state, the primary owner of forests (see Alden Wily this issue), has been constantly challenged ‘through the process of successfully defining and enforcing rights to community membership and rights of access to important resources’ as seen in the struggle over land rights. For many in the global south, while the state seeks to regulate access and exclusion from land with forests, the ideological power of markets and their framing of environmental services are becoming equally important sources of authority and legitimation (Hall *et al.* 2013). As Thompson (1975) suggests, it is around the rule of law that the future of forest and agrarian practices of the rural poor might be secured.

There are however increasing doubts as to whether the conservation of primary forest (or near-climax forests), if indeed such conservation is possible, will be sufficient to maintain the functioning of tropical forest landscapes (Chazdon 2014). In order to reach the necessary scale of vegetation cover, primary forest conservation will need to be combined with forest restoration efforts by the people who live there. This means that secondary vegetation may well become the main tropical forest cover in the future as primary forest will largely remain only on the steep, uncultivable, and inaccessible areas in the future. This requires a wider acceptance of diversity and complexity in forest forms or types (primary forest, patchy mosaic of secondary forests, and forests in agriculture) and management systems to support this, particularly of secondary growth that re-generates forest. It also brings into question the very meanings of ‘primary’ and ‘secondary’ forest as separate categories, and identifies issues of complex temporal or age interactions in forest ecology and the interaction of these with human activities.

It is widely known that large-scale deforestation of tropical forests is strongly associated with agro-capital and the expansionist tendencies of industrialised agriculture (Borras *et al.* 2016). However, there is also increasing evidence suggesting that secondary forest regeneration through smallholder action is contributing to forest recovery. Hecht (2014) describes a re-wooding of some parts of the Amazonian landscape leading to a recovery of forest area and points out that this in turn brings into question whether the agrarian can be so clearly demarcated from the forested. This emphasizes the need to broaden the lens of our understanding of agrarian change. The spread of trees into the agrarian landscape in the mid-hills of Nepal has also been observed (Marquardt *et al.* 2016). Forest regrowth can occur in many ways and there is therefore no reason to think that the variation in these forms of regrowth are any less socially or ecologically complex or independent of land use policies than are other agricultural and forestry land use practices.

Despite secondary successions being a central feature in many tropical landscapes, it is almost invisible in research as well as a low priority in policy agendas on global climate, forestry, and agriculture. Secondary forests are primarily viewed as the by-product of deforestation rather than intrinsic to forest ecology. Additionally, secondary forests are commonly thought of as being degraded, which makes it easier to justify their transformation of these forest into large-scale agriculture as opposed to opportunities to improve the livelihoods of rural people and the important role they play in the ecology of tropical forests. We believe this one-sided perception of secondary forests is problematic and is largely driven by specific expert knowledge systems in forestry, top down management, ideologies of poor farming practices and the perception of smallholders as the major drivers of deforestation. The deforestation and reforestation debates rarely consider smallholders’ practices to be based on credible knowledge systems or engage with the messy empirical reality of development where forest change outcomes reflect other complex dynamics with their own logic. As Hecht *et al.* (2014) suggested, it is often policy changes outside the forest that have the greatest effects on forest dynamics.

Yet there is a demonstrated potential in smallholders’ forest land use systems to support forest landscapes, provide livelihood security, and expand the forest area (see Alden Wily this issue). Such systems, given the constraints of family labour supply, lack the expansionist tendencies of large-scale market driven land uses. Moreover these practices vary temporally and spatially based on deep contextual knowledge of the forest, which in turn offers the possibility to secure the livelihoods of rural populations and promote forest regeneration (Pokorny 2013).

A workshop was convened in Uppsala in December 2018 to speak across disciplinary interests to these issues and explore the idea of how forest regeneration is both intrinsic to tropical forest ecology as well as an essential smallholder land use category and agricultural practice, and examine the conditions under which both might be supported. We wanted to review what we know about smallholders’ active forest regeneration management practices and investments in the landscape by drawing on and developing the notion of landesque capital (Blaikie and Brookfield 1987; Håkansson and Widgren 2014; Börjeson and Ango this issue). This requires a rethinking of what forests are and the role of smallholders in contributing to re-foresting landscapes through critical engagement with concepts of forest transition and different forms of land control. A set of questions guided the selection of papers that were presented at the workshop that this current collection is drawn from.

- What do we mean by ‘secondary forest” and how do we manage complex mosaic forest landscapes?
- What do we know about the extent of secondary forest regeneration and to what extent is it complementing and providing additional benefits (ecosystem services) to primary forest conservation?
- What has been the contribution of smallholder practices to secondary forest regeneration and what benefits do they derive from it?
- What are the legal obstacles to smallholder engagement in secondary forests and their regeneration?
- What might be a future research agenda in relation to supporting secondary forest regeneration and supporting the livelihoods of smallholder?

This introductory essay addresses the first of these questions, introduces the papers, and concludes with a discussion of the implications of the debate for future research on secondary forests.

## Going beyond the Primary Vs Secondary Dichotomy

**Fig 1:**
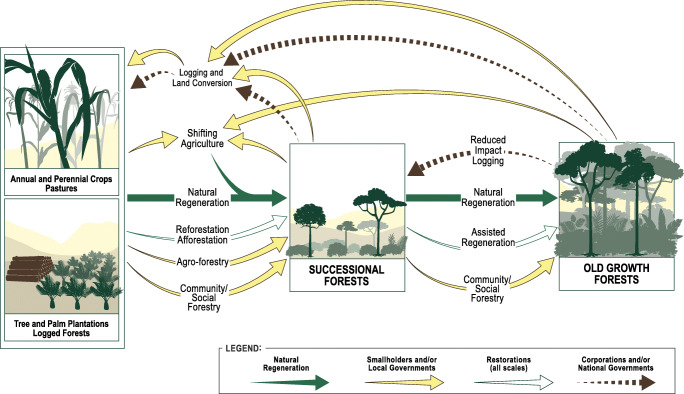
Conceptual model showing how different types of anthropogenic disturbances, management practices and successional changes influence tropical forested landscapes. For each arrow the colours indicate the main actors or processes responsible

The Food and Agriculture Organization of the United Nations (FAO) defines secondary forests as “forests regenerating, largely through natural processes, after a significant disturbance of the original forests and displaying major differences in forest structure and/or species composition compared to pristine primary forests” (FAO 2003). Roughly two thirds of the global forested area are classified as secondary forests (FAO 2010; Mackey *et al*. 2015). In the tropics, secondary forests cover ca. 70% of the entire forested area (FAO 2010) and are thus considerably more common than primary forests. Despite the growing awareness of the extent and importance of secondary forests, these assessments provide little to no information about the causation, human interactions, contribution, and governance of secondary forests. Instead, secondary forests are often all lumped together with the only unifying characteristic being that they are different from pristine, primary forests. This simple dichotomy between primary and secondary forest fails to consider the numerous and diverse natural and anthropogenic drivers influencing and shaping forested ecosystems (Fig. 1).

A consequence of this simple binary between pristine, primary forests and “less” pristine secondary forests is that secondary forests are often thought to be impaired, degraded, flawed, or defective compared to pristine forests, making it easier to justify further transforming them, for example, into plantations. It is well documented that land use changes such as deforestation or agricultural expansion can have a strong negative effect on biodiversity (Sala *et al.* 2000) with species abundance and biodiversity often reduced in secondary compared to primary forests (Mackey *et al.* 2015). However, other studies have shown few differences in total species richness between primary and secondary forests (Lawton *et al.* 1998; Barlow *et al.* 2007; Berry *et al.* 2010; Hector *et al.* 2011). Despite small changes in total species richness, these studies have shown that secondary forests are often characterized by a different range of species compared to primary forests (see Mertz *et al.* this issue). Thus, rather than being viewed as impaired, flawed, or defective in some way, secondary forests are simply a different type of forest with a different suite of species (the issue of what secondary forest should be compared to is addressed by Mertz *et al.* this issue). Nevertheless, it is also important to point out that primary forests provide specific habitat types and characteristics that are not present in secondary forests. Many species are dependent on these unique properties, and thus cannot persist without primary forests (Barlow *et al.* 2007; Gibson *et al.* 2011).

In some respects secondary forests could be viewed as having greater potential than primary forests. For example, it has been shown that secondary forests sequester atmospheric CO_2_ ten times greater than primary forests (Poorter *et al.* 2016). Chazdon *et al.* (2016a) estimated that secondary forests in Latin American can sequester 8.5 Pg C in above ground biomass during 40 years, which is equivalent to carbon emissions from fossil fuel use and industrial processes in all of Latin America and the Caribbean from 1993 to 2014. Additionally, Bastin *et al.* (2019) recently mapped the global potential for tree cover and showed that there is room for an extra 0.9 billion hectares of forest cover, which in turn could sequester 205 gigatonnes of carbon. Thus, secondary forests and global tree restoration efforts (e.g., Bonn Challenge and the New York Declaration on Forest) represent a large and important potential carbon sink for atmospheric CO_2_. Thus, the common assumption that secondary forests are impaired, degraded, or defective compared to primary forests appears not to be correct as secondary forests have the potential to play a critical role in climate mitigation.

It is commonly agreed that secondary forests are regenerating from a significant disturbance, yet what constitutes a significant disturbance, especially in terms of the intensity of the disturbance, is debatable. In many ecological textbooks, primary forests are often described as a climax community that is “stable” and thus experiences little to no disturbances (Cain *et al.* 2014; Mackenzie *et al.* 1998). However, this traditional perception of primary forests is being questioned. It is now recognized that disturbances, many of which are anthropogenic, are a core feature of forest ecosystem dynamics (e.g., ‘patch dynamics’ and the ‘non-equilibrium’ view) and thus the distinction between climax (i.e., primary) forests and secondary forests is not as obvious as originally thought (Chokkalingam and De Jong 2001). As Börjeson and Ango (this issue) point out, human disturbance can also be an investment of labour to maintain or enhance productivity and thus the antithesis of degradation. Whittaker and Levin (1977) argue that the vegetation on the Earth’s surface is in constant flux and what we observe is not simply successions and climax communities, but instead a mosaic of plant communities existing in different kinds and degrees of stability and instability. This has led to a growing consensus that the classical definition of primary forests as those that do not experience disturbances and therefore represent a stable climax community may be misleading. Instead, disturbances, especially anthropogenic disturbances, play an important role in both primary and secondary forest dynamics, raising the broader question of causes and consequences of human intervention in forested ecosystems.

In order to go beyond the simple dichotomy of primary vs. secondary forest, it is necessary to identify and understand the main drivers of forest loss leading to secondary forests and the implications of these for forest ecosystems (Chokkalingam and De Jong 2001). It should be remembered that the main drivers of forest loss can be anthropogenic as well as natural disturbances although there are methodological and data challenges in attributing forest loss to specific causes. In temperate and boreal forests Curtis *et al.* (2018) assess the main cause of forest loss to be from natural wildfires and forest management practices. In contrast, in addition to wildfires they consider the biggest threat to tropical forests to be human intervention through commodity-driven deforestation, row crop agriculture, and cattle grazing in South America, oil palm plantations in Southeast Asia, and shifting and subsistence agriculture by indigenous people in Africa (ibid.). It is important to recognize and understand the numerous drivers leading to secondary forests as they highlight the range of different ways in which humans interact with forests. These in turn are strongly coupled to people’s livelihoods, financial incentives, and the tenure regimes in which forests are embedded.

Central to understanding forest dynamics is the question of who has control over forest land and how that control is exercised, crucially, how territory is defined and by whom, who regulates the use of that territory, and the property or ownership rules that are imposed (individual, community, state). As Alden Wily (this issue) makes clear, the assumption that the state is the best defender of forests is under critical attack and she proposes a different vision for the future of tropical forests in which the emerging trend of community-owned forest land provides both use values and protection. In the global south, local forest user groups’ views on what constitutes territory and who has authority over territory have often been at variance with forest department or state views, leading to a long history of contestation (as in Nepal and Peru, see Pain *et al. *2020). Moreover, where the state has less regulatory authority for reasons of weakness and/or collusion with powerful national or regional forest companies or agribusinesses and where forest boundaries are not clearly defined, deforestation may be aggressive and destructive.

There is also a need to recognise the drivers outside forests linked to agrarian transitions that have impacts on forest cover and use. There are diverse routes to forest transition (the determination of the forest boundary and forest recovery) and their relation to agrarian transitions are likely have a strong influence on tropical forest landscapes. Intensification and market engagement can result in greater deforestation. Securing household subsistence can also follow a tree management intensification route, as has been observed in Nepal (Pain *et al.* 2020), but equally plantation agriculture can lead to forest substitution. Lastly, global policies in relation to forest conservation can impact tree management practices both within and outside forests (ibid.). These diverse processes can all be operating at the same time leading to contradictory impacts and sometimes perverse outcomes.

In summary, when characterizing secondary forests, it is essential to account for the regulatory framework surrounding secondary forest, the security of property rights, and the diverse drivers of agrarian and forest change.

## Rethinking the Forest Landscape

### The Schema

Given inconsistencies in definitions as well as increasing human pressure on forest ecosystems we do not see any value in continuing to characterize forests simply as either primary or secondary. We argue that there is a need to rethink how forested landscapes are characterized and propose a new conceptual model to do this.

Figure 1 presents our schema, an approach that takes a more discriminating approach than a simple primary-secondary binary classification of forests. It directs attention to how governments on different scales, corporations, and smallholders influence the forested landscape through different types of anthropogenic disturbances and management practices. Reading from left to right the figure represents successional changes after a disturbance, with logged forests or deforested landscapes on the left and old-growth, more mature forests on the right. The various line colours highlight the main actors or processes active in the landscapes and each arrow is tied to a certain kind of anthropogenic disturbance or management practice observed in tropical forests. Restoration can be done on all scales and by all actors, and natural regeneration occurs automatically when no actors are present. This aids more analytical thinking about the diversity of secondary forest formations and their implications for future forests.

### Disturbances

In our conceptual model, we acknowledge the importance of both natural and anthropogenic disturbances in forested landscapes but mainly focus on the importance of anthropogenic disturbance. As is the case with natural disturbances, anthropogenic disturbances vary in their causation and severity, ranging from small, local disturbances to larger scale deforestation and land degradation that can occur across a range of temporal scales. We must also remember that such disturbances by design can contribute to long-term land productivity enhancement (see Börjeson and Ango this issue). We further suggest that there is a correlation between land-tenure and financial incentives and the severity of the disturbance. For example, many indigenous people, who often lack land-tenure, practice shifting and subsistence agriculture to sustain their livelihoods. Although these practices represent a disturbance to forest landscapes, these disturbances are often for subsistence living, occur at small spatial scales, and do not necessarily cause large-scale degradation. However, if the frequency of these small-scale disturbances is high, forests may not be able to recover, resulting in cumulative and potentially large and negative effects on forest landscapes. In contrast, government agencies and/or large private companies, which often own large amounts of land, tend to manage the land in a way to maximize their financial gains. In doing so, forested landscapes are often drastically altered at large spatial scales, as can be seen in the conversion of tropical forest to tree plantations. Not surprisingly, financial incentives and the nature of land-tenure regimes (see Alden Wily this issue) are the main drivers shaping tropical forested landscapes.

### Regeneration

It is commonly assumed that, if secondary forests are undisturbed by recurrent disturbances, with enough time they will revert to pristine, primary forests (Brown and Lugo 1990; Corlett 1994). But this assumption ignores the investment of human labor to support regeneration processes (see Börjeson and Ango this issue), which is often the case when the disturbance is relatively small scale (see Pain *et al.* 2020). This human intervention can serve to selectively enrich natural secondary forest (see Peru, ibid.) or lead to significant change in forest species composition (see Nepal, ibid., and the coffee plantations in Ethiopia, Börjeson and Ango this issue). Large-scale disturbance may also result in an entirely different floristic composition, as is the case of the secondary babassu forests in Maranhão, Brazil (Porro and Porro 2014).

In many places, forest restoration may be needed to recreate the original structure and biodiversity associated with primary forests (Chazdon 2008). The possibility of restoration greatly depends on the initial state of the forest and the intensity of disturbance and degradation and can be extremely timely and costly. Moreover, there has recently been a growing number of afforestation initiatives (e.g., Bonn Challenge, Regreening Africa, and the New York Declaration on Forest) with the goal of curbing land degradation and assisting in climate mitigation. Consequently, human intervention is now playing a critical role in the recovery of forested landscapes, although differences in law and governance practices are likely to strongly influence outcomes.

### Public Authority and Secondary Forests

The control over forest land is ultimately what determines how forests are managed (the management regime) and by whom. Central to land control is how territory is defined (Hall 2013), who regulates the use of that territory and the property or ownership rules that regulate land use. Territory is land that is considered as belonging to a particular person, people or country. In the global south there is rarely one single source of authority over the regulatory control of forest land and consequently forest territory is often contested. In such cases, there is a need to better understand the processes of mobilisation of territorial control and authority over its use (see Arora-Jonsson *et al.* this issue).

In many contexts governments have limited control over its territory and authority and legitimacy are often questioned (see Peru, Pain *et al.* 2020). This creates a disjuncture between a state’s ‘de jure’ sovereignty – which establishes the state’s boundaries and the state’s authority to control society within these borders through international law – and its ‘empirical’ sovereignty, which is its ability to actually impose its authority over territory (Lund 2011). In many tropical countries there is often ‘fragmented’ sovereignty and attempts to establish territorial control, not least over the forests and people, are not simply the result of extending formal government structures. Rather, the extent and degree of control are shaped by forms of competition and coalition between a wide range of formal and non-formal actors, including government departments, private companies, non-government organisations, distinct social groups, and possibly, armed groups.

A central question then with respect to secondary forest landscapes is how public authority is established and contested and how this is reflected in the ways that the forest economies are managed and regulated. Analysing the nature of public authority can further understanding of the composite and partial forms of governance, the spatial control and territorialisation of the forests, and the nature of secondary forest formation and regeneration. Thus, shifting agriculture is more likely to take place at a small scale where government authority is limited. Conversely, large-scale timber plantations may reflect stronger government presence while large-scale commercial crop plantations that result from forest clearance may reflect private companies’ influence over government authority.

Central to the issue of public authority and its exercise are those of rights (Lund 2016). The key struggles are those over the ability of forest-based populations to secure property rights (see Alden Wily this issue) and the inability of states to guarantee property rights, either collectively or individually, reflecting their lack of authority and legitimacy. As Lund (2016:1) notes: ‘rights do not simply flow from authority but also constitute it. Authority and rights are conceptually tied together by recognition.’

#### Review of Papers in Collection

The five papers that follow this introduction develop and elaborate on specific aspects of our schema. Pain *et al.*, through an analytical contrast of the role and contribution of secondary forest in the Nepalese mid-hills and Peruvian Amazon, provide an empirical foundation to the schema and to the papers that follow. They draw attention to the diverse forms of secondary forest in the two countries, the conditions under which they arose and the interplay between forest dynamics, governance regimes, and wider agrarian processes. While Nepal is primarily an account of the significance of secondary forest and the role of community forestry, since most primary forest is long gone, the Peruvian Amazon clearly illustrates diverse forms of secondary forests, how they are created, and the consequences of a forestry agenda that favours primary forest and conservation over secondary forest formations.

Börjeson and Ango build on Pain *et al.*, speaking to the contribution of smallholders to secondary forest regeneration, and show the investment of human labour needs to be taken into account during the creation of secondary forest landscapes. Conceptually they draw on the notion of landesque capital (Blaikie and Brookfield 1987), and specifically “green” landesque capital to emphasize the co-work of nature and labour in the regeneration of tropical forests. They illustrate Landesque capital, conceptualised as an investment of site specific resources and labour for future production, and the relative contribution of human labour and nature through a case study of different forest mosaic landscapes in South-Western Ethiopia where coffee is an important cash crop.

Arora-Jonsson *et al.* move beyond the dominant narrative of forests being about trees and unpack the social life of forests to explore, in particular, issues of gender and violence in forests. Forests have long been framed as sites of men’s work and values neglecting both the work in kind and directly that women do in forest management. Violence is an element of daily life in the Peruvian forest (see Pain *et al.*) and, like forest change processes, can only be understood by reference to wider agrarian processes of change. Site specific violence within the forest and forest families has to be understood as being shaped by wider frames of violence, both in terms of the structure of power, as in the denial of indigenous rights accorded by law in the case of Peru, and deep seated norms of masculinity.

As was noted by Pain *et al.*, the official forestry discourse in Peru is that the swidden agriculture practiced by rural people in the Amazon is the main cause of forest degradation and loss. But, returning us to the first two research questions that connect these papers, Mertz *et al.* review ecosystem services (ES) from secondary forests in shifting cultivation. They find that the body of evidence that robustly compared ES provision from secondary forest with comparable land uses is surprisingly thin and limited. Moreover, much of the evidence they review is equivocal, based on contrasts of a very restricted number of ES. They also raise issues of how comparable ES are between secondary and primary forests. A more inclusive perspective on ES, including food provisioning and cultural ecosystem services would probably place secondary forest in a more favourable light.

Finally, Alden Wily addresses the central issue of the legal framework and territorial rights accorded to forest users. She makes a strong case, in many ways backed by current trends in giving communities tenure rights over their forests, that giving communities secure property rights through forms of collective tenure is necessary to secure forest futures. Neither state ownership nor commercial capital have shown themselves to be interested or competent guardians of the forests and there is increasing recognition community based governance has more to offer.

#### Towards a Future Research Agenda

Most research on “secondary forests” has been from an ecological perspective, focussing primarily on a restricted range of environmental services, notably biodiversity and carbon sequestration. However, our new conceptual model highlights the importance of human intervention in shaping tropical forest landscapes, and thus we believe a key question that needs to be further addressed is how different types of human intervention and the various scales at which they act influence forested ecosystems. It is also crucial to better understand how these altered forested landscapes affect humans and societies (e.g., health, gender equality, migration). Despite previous studies comparing biodiversity and carbon sequestration between primary and secondary forests, we know less about how ES services vary among different types of secondary forests, not only with respect to biodiversity and carbon sequestration, but also other less studied ES. One forest ES service that may be particularly important and linked to biodiversity, as the current Covid 19 pandemic illustrates in terms of the rising risks of pandemics (Tolleson 2020), is effects (or disservices) on human health and how that varies among different forested ecosystems. A further dimension to this might be the risk of human diseases (e.g., malaria) among different forest landscapes. A better understanding of how ES vary among forested landscapes is essential as many rural people in the tropics rely on forests for their livelihoods. This would also move the relations on ecosystem services and forests into more of a landscape perspective that contains diverse land uses rather than confining it to forest ecosystems alone (Chazdon *et al.* 2016b).

But central to a future research agenda is learning more about what drives the shaping of authority and rights in forest and land practices (policies, programmes, land tenure arrangements) and the management of disturbance in forests. More needs to be known of the links between disturbance regimes and the consequences of these at different scales for the various types of successional forests as well as the people who use them for their livelihoods. Given the diverse and gender-based interests of different categories of smallholders, groups, and other interested parties, more understanding is needed as to how they perceive and interpret policies regarding land use, land ownership, and preservation of forests areas: what their forms of resistance and tactics of engagement with the state are, particularly with regard to territorial rights and how do they differ among different successional forests. This will have consequences in terms of how people interact with forests and how they influence the form and complexities of forest landscapes, particularly where successional and managed forests replace previous old growth rainforests. In turn, all the above processes will not only strongly influence the regeneration of tropical forests but will have consequences in shaping the wellbeing of individuals relying on tropical forests for the livelihoods.
